# Prevalence and clinical implications of respiratory viruses in asthma during stable disease state and acute attacks: Protocol for a meta-analysis

**DOI:** 10.1371/journal.pone.0294416

**Published:** 2023-11-15

**Authors:** Gioulinta S. Alimani, Sachin Ananth, Cristina Boccabella, Ekaterina Khaleva, Graham Roberts, Nikolaos G. Papadopoulos, Chris Kosmidis, Jørgen Vestbo, Effie Papageorgiou, Apostolos Beloukas, Alexander G. Mathioudakis

**Affiliations:** 1 Department of Biomedical Sciences, University of West Attica, Athens, Greece; 2 London North West University Healthcare Trust, London, United Kingdom; 3 Department of Cardiovascular and Thoracic Sciences, Fondazione Policlinico Universitario "A Gemelli"—IRCCS, University of the Sacred Heart, Rome, Italy; 4 Faculty of Medicine, Clinical and Experimental Sciences and Human Development in Health, University of Southampton, Southampton, United Kingdom; 5 Paediatric Allergy and Respiratory Medicine, University Hospital Southampton NHS Foundation Trust, Southampton, United Kingdom; 6 Division of Immunology, Immunity to Infection and Respiratory Medicine, School of Biological Sciences, The University of Manchester, Manchester Academic Health Science Centre, Manchester, United Kingdom; 7 Allergy Department, 2nd Paediatric Clinic, National and Kapodistrian University of Athens, Athens, Greece; 8 Division of Evolution, Infection and Genomics, School of Biological Sciences, The University of Manchester, Manchester Academic Health Science Centre, Manchester, United Kingdom; 9 The North West Lung Centre, Wythenshawe Hospital, Manchester University NHS Foundation Trust, Manchester, United Kingdom; 10 National AIDS Reference Centre of Southern Greece, University of West Attica, Athens, Greece; Fiocruz Mato Grosso do Sul, BRAZIL

## Abstract

**Introduction:**

Viruses are detected in over 50% of acute asthma attacks and in a notable proportion of patients with asthma during stable disease state They are associated with worse outcomes. We will conduct a series of systematic reviews and meta-analyses to quantify the prevalence and clinical burden of various respiratory viruses in stable asthma and acute asthma attacks. In addition, we will assess the viral loads of respiratory viruses during stable and acute asthma, to explore whether viral load could differentiate attacks triggered by viruses versus those where viruses are present as “innocent bystanders”.

**Materials and methods:**

Based on a prospectively registered protocol (PROSPERO, ID: CRD42023375108) and following standard methodology recommended by Cochrane, we will systematically search Medline/PubMed, EMBASE, the Cochrane Library and relevant conference proceedings for studies assessing the prevalence or clinical burden of respiratory viruses in asthma. Methodological rigour of the included studies will be appraised using a tool specific for prevalence studies and the Newcastle-Ottawa Scale respectively. In anticipation of significant clinical and methodological heterogeneity, we will conduct random effect meta-analyses. For evaluating the prevalence of viruses, we will perform meta-analyses of proportions using the inverse variance method, and the Freeman-Tukey transformation. We will conduct meta-regression analyses for exploring heterogeneity.

**Conclusion:**

We envisage that these systematic reviews and meta-analyses will quantify the prevalence and burden of respiratory viruses in stable and acute asthma and will drive future research and clinical practice.

## Introduction

Despite significant progress in the diagnosis, assessment, and management of asthma, it is still associated with important morbidity and unacceptable mortality [1,2]. In addition, it imposes a considerable economic burden on healthcare systems and the society [1–3]. Asthma is characterised by chronic airway inflammation and hyperresponsiveness that cause chronic respiratory symptoms, mainly breathlessness, wheeze, and cough [1,2]. In addition, patients experience acute attacks that are characterised by pronounced symptoms and often lead to emergency presentations and/or hospitalisations, as well as absence at work [1,2].

Respiratory viruses are identified in up to 90% of asthma attacks [4–10]. They are also detected in a notable proportion of patients during stable disease state, although their prevalence has not been formally assessed in a meta-analysis. However, an observational study reported the detection of adenovirus and rhinovirus in 78.4% and 32.4% of unselected children with well controlled asthma [11]. The presence of respiratory viruses during stable disease state is predictive of poorer asthma control, more frequent attacks, and reduced lung function [12,13]. While the undifferentiated presence of any virus in the airways is not associated with clinical severity or recovery of acute attacks, the presence of specific viruses has been associated with adverse outcomes [14]. For example, human rhinovirus-A has been associated with a longer duration of symptoms during asthma attacks [14]. These observations suggest differences in the characteristics and severity of asthma attacks triggered by different viruses and virally induced immune pathways [15]. In parallel, it is not clear whether viruses detected in the respiratory tract during an acute event are necessary driving the asthma attack, or whether they may simply be an incidental finding (innocent bystander), in view of their high prevalence during stable disease state.

A higher viral replication would be anticipated during an acute viral infection. Therefore, viral load could potentially distinguish between attacks triggered by viruses and those where the virus is a bystander, if it is assessed during the very early stage of the attack [16]. Indeed, a prospective observational study revealed higher viral loads in patients with asthma during an attack compared to stable disease state [17]. Similar findings were reported in another cohort evaluating human rhinovirus in children with asthma [18]. However, these findings are not consistently corroborated in other studies [19]. A rigorous meta-analysis of all available data could assess whether the viral load of the most frequently identified respiratory viruses differ during acute attacks versus stable asthma and can thus differentiate attacks triggered by viruses, cases where the virus is an “innocent bystander”. Accurate identification of asthma attacks triggered by viruses could navigate the use of antivirals that could improve patients’ outcomes. Indeed, oseltamivir has been found to improve lung function and reduce the frequency of future attacks in children with asthma [20].

Overall better understanding is needed of the prevalence and clinical burden of respiratory viruses in stable and acute asthma. We describe a protocol for three systematic reviews aimed at evaluating: (A) the prevalence of respiratory viruses in patients with stable and acute asthma; (B) differences in the viral loads of respiratory viruses detected during stable versus acute asthma, to explore whether viral loads of prevalent respiratory viruses could be used as a means of diagnosing of attacks caused by respiratory viruses; and (C) the association between the presence of respiratory viruses and clinical outcomes of patients with stable and acute asthma.

## Materials and methods

This series of systematic reviews and meta-analyses will be based on a registered protocol at the International Prospective Register of Systematic Reviews (PROSPERO, ID: CRD42023375108). It will use standard methodology recommended by the Cochrane Collaboration and the Preferred Reported Items for Systematic Reviews and Meta-Analyses (PRISMA) [21,22]. This protocol adheres to the PRISMA extension for systematic review and meta-analysis protocols [23].

### Eligibility criteria

We will include studies evaluating the presence (prevalence) of respiratory viruses in patients diagnosed with asthma, either during stable disease or during acute attacks. We will accept studies assessing severe acute attacks requiring hospitalisation, as well as those assessing a broader population with acute attacks not necessarily requiring hospitalisation. These two subgroups will be evaluated separately. We will separately assess studies of adults versus adolescents or children aged over 5 years. Eligible studies will include patients with a clinical diagnosis of asthma consistent with national or international clinical guidelines. We will only include studies using molecular techniques to detect respiratory viruses, provided that participants are sampled prior to the initiation of any antiviral treatments. We will exclude studies focusing on specific populations who are at high risk of viral infections, such as immunocompromised patients or lung transplant recipients. We will exclude studies conducted during the coronavirus disease 2019 pandemic (2020–2022) from systematic review A, to avoid confounding, since changes in the lives of patients with asthma, such as shielding and the use of face masks have significantly affected their clinical characteristics and viral exposures [24,25].

For systematic reviews A and B, we will assess studies exploring the prevalence of respiratory viruses in patients with stable or acute asthma. For systematic review C, we will assess studies of the associations between the presence and/ or viral load of respiratory viruses and relevant asthma clinical outcomes.

We will include data both from observational and interventional studies, provided that they meet the aforementioned eligibility criteria.

### Outcomes

Systematic review A: The primary outcome will be the prevalence of each respiratory virus evaluated during stable and acute asthma. We will also consider the prevalence of any virus in studies evaluating at least three viruses, including rhinovirus.

Secondary outcomes will include:

- Seasonal variability in the prevalence and viral loads of respiratory viruses
- Percentage of patients testing positive for more than one virus.

Systematic review B: The primary outcome of this project will be mean viral loads of respiratory viruses during stable state and acute attacks.

Secondary outcomes will include the seasonal variability in the viral loads of respiratory viruses during stable asthma and acute attacks.

Systematic review C: Only studies assessing associations between the presence and/or load of respiratory viruses and clinical outcomes will be included in this project. For studies evaluating stable asthma, the primary outcomes will be:

- Annual rate of severe attacks (requiring hospitalisation due to asthma).
- Annual rate of moderate or severe attacks (requiring systemic steroid treatment or an emergency visit due to asthma)
- Mortality

Secondary outcomes will include:

- Patient reported symptom severity
- Exercise capacity
- Forced expiratory volume in 1 second (FEV_1_) decline rate

For studies evaluating attacks of asthma, primary outcomes will include:

- Mortality
- Treatment success/ failure rate. We will use each individual study’s definition of treatment success or failure when assessing treatment this outcome. In acute respiratory diseases, treatment failure is usually defined as a composite outcome consisting of several adverse outcomes such as lack of clinical improvement, need for additional treatment, need for ICU admission, or death, together describing an overall unfavourable outcome [26,27]. Treatment success is either defined as a composite or descriptive outcome. The latter is based on a qualitative or semi-quantitative descriptions to describe cure, such as complete symptoms resolution or adequate symptoms improvement, such that no additional treatment is deemed necessary [27].

Secondary outcomes will be:

- Symptom severity and duration
- Length of hospitalisation
- Frequency of attack recurrence and time-to-next attack
- Percentage of patients who received antiviral treatment
- Co-existing bacterial infection.

All clinical outcomes will be evaluated at the longest follow-up point. The treatment failure and treatment success rates in studies assessing acute attacks will be evaluated at 1–4 weeks from presentation.

### Systematic literature search

We will systematically search Medline/PubMed, EMBASE and the Cochrane Library to identify randomized controlled trials and observational studies evaluating the prevalence and clinical implications of respiratory viruses in stable and acute asthma. The electronic databases will be searched in May 2023. We will also search the World Health Organization International Clinical Trials Registry Platform (ICTRP) search portal, the abstract proceedings of the European Respiratory Society, the American Thoracic Society, the Asian Pacific Society of Respirology, the European Society of Clinical Microbiology and Infectious Diseases, the American Society of Microbiology, the European Society for Clinical Virology, the European Academy of Allergy and Clinical Immunology, the American Academy of Allergy, Asthma and Immunology, and the World Allergy Organization, as well as the reference lists of all included studies and all previously published systematic reviews. This strategy was developed by one author (AGM) and was refined after the identification of Medical Subject Headings (MeSH) terms from eligible studies that were identified during pilot searches. Detailed search strategies are available in S1 File. All sources will be searched from inception, without language limitations and time restrictions. Two investigators will independently screen the titles and abstracts of all studies that our searches will identify. The full-text versions of all potentially eligible manuscripts and abstracts will then be acquired and reviewed for confirmation by two authors independently. Disagreement in any step of the systematic review process will be resolved by discussion with a third investigator.

### Data extraction

One reviewer will extract data from each eligible study using a pre-defined, pilot-tested spreadsheet, with verification by a second reviewer. The full reference of each eligible study, as well as details on the study design, eligibility criteria, baseline characteristics, details on the viruses evaluated and the validation of laboratory assays used for viral identification and/or quantification, will be extracted by one reviewer and will be cross-checked by another reviewer for validity. Data regarding the prevalence and viral loads of respiratory viruses and all clinical outcomes will be extracted by two reviewers. Details on all data and variables that will be extracted are available in S1 File. Variables may be added to data extraction following discussion between the reviewers. Missing data may be requested from the study investigators.

### Risk of bias

For systematic reviews A and B, we will use the risk of bias tool for prevalence studies that was developed by Hoy et al [28]. For systematic review C, we will use the Newcastle-Ottawa Scale [29]. Both these tools rigorously assess the representativeness of the study participants, which is crucial to our work. Risk of bias will be assessed by two investigators independently, and will be reported for each of the domains proposed by the assessment tools. Furthermore, an overall risk of bias judgement will be made for each study.

### Unit of analysis

For studies evaluating stable asthma, we will accept re-evaluation of the same patients (study re-entry) but only if the interval is at least 3 months. For studies assessing asthma attacks, the unit of analysis will be the attack, rather than the patient; therefore, we will include the first assessment of respiratory viruses during each attack.

### Data synthesis

Study heterogeneity will be measured using the I^2^ statistic. Substantial heterogeneity (I^2^ > 50%) will be investigated using meta-regression analyses. In anticipation of significant clinical and methodological heterogeneity, we will conduct random effect meta-analyses. For evaluating the prevalence of viruses, we will perform meta-analyses of proportions using the inverse variance method, and the Freeman-Tukey (double arcsine) transformation [30,31]. This method addresses variance instability, as well as the problem of confidence intervals falling outside the 0–1 range, both important issues in meta-analyses of the less prevalent viruses [31]. For assessing publication bias, we will produce funnel plots with sample size as the measure of accuracy, which are more appropriate for meta-analyses of proportions [32].

### Meta-regression and sensitivity analyses

If sufficient data are available, we will perform univariate meta-regression analyses followed by stepwise multivariate meta-regression analysis to evaluate the impact of the following parameters on the outcomes:

1. The season when the samples were collected, or at least the proportion of samples that were collected during the influenza season.
2. The year that the study was completed
3. The percentage of patients that were vaccinated for influenza
4. Concomitant use of inhaled corticosteroids
5. GINA asthma severity [2]
6. Use of antiviral treatment (Systematic review C only)

Moreover, we will conduct the following sensitivity analyses:

- We will only include studies at a low risk of bias.
- We will repeat meta-analyses using a fixed-effects model.

### Certainty of evidence

Systematic reviews A and B: Unfortunately, there are no validated tools for assessing the certainty in a body of evidence around prevalence studies. However, we will comment in our report on the representativeness of the participants of the included cohorts, overall risk of bias, heterogeneity of data, and whether that was resolved through the planned meta-regression analysis.

Systematic review C: For each outcome, we will evaluate the quality of the body of evidence using the GRADE methodology (Grading of Recommendations Assessment, Development and Evaluation). GRADE, which is based on the methodological quality, confounders, and publication bias, assesses the confidence in a body of evidence as high, moderate, low or very low [33].

### Protocol deviations

Any deviations from this published protocol will be documented and justified in our final report.

### Patient and public involvement

This systematic review protocol was developed without patient or public involvement.

## Ethics and dissemination

Ethical approval is not required for this systematic review and meta-analysis of published, aggregate data.

The results of this systematic review will be presented in national and international conferences and will be published in high-impact peer review journals.

## Discussion

We describe the study protocol of a series of systematic reviews and meta-analyses that will assess the prevalence and clinical burden of respiratory viruses and stable and acute asthma. The results of this meta-analyses are anticipated to drive future research and policy.

The prevalence of respiratory viruses in asthma attacks has been evaluated previously in two systematic reviews [4,5,10]. The latest literature review was conducted in 2014 and our study will update these systematic reviews [4,5,10]. In addition, our planned systematic searches are broader and likely to identify additional studies. More importantly, a central objective of our work is to compare the prevalence of respiratory viruses during acute versus stable asthma and to our knowledge no previous systematic review has addressed the latter. Moreover, viral loads or the clinical burden of respiratory viruses in patients with asthma have not been evaluated in an evidence synthesis.

We expect our work will have important clinical and research implications. Respiratory viruses associated with very mild infections in otherwise healthy people, such as rhinovirus, appear to be associated with more severe presentations among patients with asthma [14] and, in this setting, antiviral treatment may be more beneficial. Therefore, our results are expected to drive clinical research and practice around the use of antivirals for selected asthma attacks. In parallel, our group is conducting similar work among patients with COPD and we will be able to explore for differences in the viral patterns across the two diseases [34].

These systematic reviews have important strengths. They will be based on broad screening of three large online databases and relevant literature. In addition, we will use rigorous methodology for appraising the available data and for conducting state-of-art meta-analyses and meta-regression analyses. We anticipate significant methodological and clinical heterogeneity in the included studies, but we hope that we will be able to explain this heterogeneity through the planned meta-regression analyses. Moreover, quantification of viral loads is not standardised yet and combining data in meta-analyses may be challenging.

Characterisation and prognostication of asthma and acute asthma phenotypes, to facilitate the introduction of precision medicine interventions is consistently highlighted as a research priority by relevant stakeholders [35,36].

Overall, we aspire that these systematic reviews and meta-analyses will drive future research and clinical practice. We envisage that this may clarify if antivirals should have a more important role in the management of asthma attacks.

## Supporting information

S1 Checklist PRISMA-P (Preferred Reporting Items for Systematic review and Meta-Analysis Protocols) 2015 checklist: Recommended items to address in a systematic review protocol*. (DOC)

S1 File Section S1: Search strategies. Section S2: Data extractions, variables to be captured.
(DOCX)
