# Supplementary material for: Prevalence and clinical implications of respiratory viruses in asthma during stable disease state and acute attacks: Protocol for a meta-analysis
Source: PLoS One. 2023 Nov 15;18(11):e0294416. doi: 10.1371/journal.pone.0294416 (PMC10651012; doi:10.1371/journal.pone.0294416)
Supplement: S1 File — Section S2: Data extractions, variables to be captured. (DOCX) [file pone.0294416.s002.docx]

**Online appendix**

Prevalence and clinical implications of respiratory viruses in asthma during stable disease state and exacerbations: Protocol for a meta-analysis

Gioulinta S. Alimani, Sachin Ananth, Cristina Boccabella, Ekaterina Khaleva, Graham Roberts, Nikolaos G. Papadopoulos, Chris Kosmidis, Jørgen Vestbo, Effie Papageorgiou, Apostolos Beloukas, Alexander G. Mathioudakis.

**Section S1: Search strategies**

**Search Strategy - Medline, PubMed & Cochrane Library**

#1 Asthma [MH]

#2 Lung Diseases, Obstructive [MH:NOEXP]

#3 Anti-asthmatic agents [MH]

#4 Asthma [tiab]

#5 Bronchospas* [tiab]

#6 Bronchoconstrict* [tiab]

#7 Bronch* [tiab] and (constrict* [tiab])

#8 (bronchial* [tiab] or (respiratory[tiab]) or (airway* [tiab]) or (lung* [tiab])) and (hypersensitiv* [tiab] or (hyperreactiv* [tiab]) or (allerg* [tiab]))

#9 OR / 1-8

#10 Viruses[MH]

#11 Influenza, Human[MH]

#12 Rhinovirus[MH]

#13 Respiratory Syncytial Viruses[MH]

#14 Coronavirus[MH]

#15 Paramyxoviridae Infections[MH]

#16 Orthomyxoviridae[MH]

#17 Adenoviridae [MH]

#18 Picornaviridae[MH]

#19 Metapneumovirus[MH]

#20 Enterovirus[MH]

#21 Cytomegalovirus[MH]

#22 Herpesvirus 3, Human[MH]

#23 Bocavirus [MH]

#24 Erbovirus [MH]

#25 Virus*[tiab]

#26 Viral*[tiab]

#27 Influenza*[tiab]

#28 Rhinovir*[tiab]

#29 Respiratory Syncytial Vir*[tiab]

#30 Coronavir*[tiab]

#31 Paramyxovir*[tiab]

#32 Orthomyxovir*[tiab]

#33 Adenovir*[tiab]

#34 Picornavir*[tiab]

#35 Metapneumov*[tiab]

#36 vzv[tiab]

#37 varicella[tiab]

#38 Enterovir*[tiab]

#39 Parainfluenza[tiab]

#40 Echovir*[tiab]

#41 SARS [tiab]

#42 MERS [tiab]

#43 SARS-CoV2[tiab]

#44 Erbovirus [tiab]

#45 Bocavirus [tiab]

#46 Polyomavirus [tiab]

#47 Middle east respiratory syndrome [tiab]

#48 Severe acute respiratory syndrome [tiab]

#49 OR / 10-48

#50 Animals[mh] not (humans[mh])

#51 editorial[publication type]

#52 review[publication type] not (systematic review [publication type])

#53 Letter [publication type]

#54 case report [ti]

#55 report of a case [ti]

#56 a case of [ti]

#57 (mice[ti] or (mouse[ti]) or (rat[ti]) or (animal*[ti]) or (pig[ti]))

#58 ((COPD[ti]) or (chronic bronchitis[ti]) or (emphysema[ti]) or (chronic obstructive pulmonary disease [ti]) or (cancer [ti]) or (bronchiectasis[ti]) or (fibrosis[ti]) or (surgery [ti]) or (operation[ti])) not (asthma[ti])

#59 OR / 50-58

#60 #9 and #49

#61 #60 NOT #59

**Search Strategy: EMBASE**

#1 exp Asthma/

#2 exp Lung diseases, obstructive/

#3 exp Allergic airway/

#4 asthma.tw.

#5 #1 or #2 or #3 or #4

#6 exp virus/

#7 exp influenza/

#8 exp rhinovirus/

#9 exp Respiratory Syncytial Virus/

#10 exp Coronavirus/

#11 exp Paramyxoviridae Infections/

#12 exp Orthomyxoviridae/

#13 exp Adenoviridae/

#14 exp Picornaviridae/

#15 exp Metapneumovirus/

#16 exp Enterovirus/

#17 exp Cytomegalovirus/

#18 exp bocavirus/

#19 exp erbovirus/

#20 virus$.tw.

#21 viral$.tw.

#22 Influenza$.tw.

#23 (("haemophilus influenzae" or "h. influenza") not influenza).tw.

#24 #22 not #23

#25 Rhinovir$.tw.

#26 Respiratory Syncytial Vir$.tw.

#27 Coronavir$.tw.

#28 Paramyxovir$.tw.

#29 Orthomyxovir$.tw.

#30 Adenovir$.tw.

#31 Picornavir$.tw.

#32 Metapneumov$.tw.

#33 Enterovir$.tw.

#34 Parainfluenza.tw.

#35 Echovir$.tw.

#36 Bocavir$.tw.

#37 vzv.tw.

#38 varicella.tw.

#39 MERS.tw and virus.tw

#40 SARS.tw and virus.tw

#41 middle east respiratory syndrome.tw

#42 severe acute respiratory syndrome.tw

#43 #6 or #7 or #8 or #9 or #10 or #11 or #12 or #13 or #14 or #15 or #16 or #17 or #18 or #19 or #20 or #21 or #24 or #25 or #26 or #27 or #28 or #29 or #30 or #31 or #32 or #33 or #34 or #35 or #36 or #37 or #38 or #39 or #40 or #41 or #42 or #43

#44 exp animals/ not exp humans/

#45 exp editorial/ or (exp review/ not (exp systematic review/))

#46 COVID-19.ti.

#47 Coronavirus.ti and 2019.ti

#48 SARS-cov2.ti.

#49 SARS-CoV-2.ti.

#50 #5 and #43

#51 #50 not (#44 or #45 or #46 or #47 or #48 or #49)

**Section S2: Data extractions, variables to be captured.**

*Study characteristics - General:* study ID, study/cohort name, study registration number, full references of all citations referring to the study, year of publication, country(-ies), continent(s), number of recruiting centres, study design, study setting.

*Description of the study population:* Stable asthma or exacerbations, general or specific population tested, total number of participants evaluated during stable disease, total number of participants evaluated during exacerbation, age, sex, smoking history, sampling period, %sampled during the flu season [October-May], GINA asthma stage, history of exacerbations, respiratory symptoms [mMRC, CAT], use of ICS (%), severity of the index exacerbation, >1 exacerbation allowed per participant.

*Risk of bias assessment:* The relevant risk of bias tool(s) will be completed for each included study.

*Respiratory viruses:* Type of sample, number of viruses evaluated, number of patients testing positive for any virus, number of patients tested for any virus, number of patients testing positive for more than one virus, number of patients testing positive for each virus type or subtype tested, number of patients that were tested in each case, data on the viral loads of each virus, assay used (commercial versus in-house; adequately validated).

*Clinical outcomes:* Data on each of the selected outcome measures will be extracted. These will include a detailed description of the definition of the outcome, the effect estimates and confidence intervals, as well as the timing of every measurement. Outcome measures in relevant subgroups of participants will also be extracted.

Data collected during stable disease versus exacerbations will be clearly separated.
